# Sensitivity Analysis of Sensors in a Hydraulic Condition Monitoring System Using CNN Models

**DOI:** 10.3390/s20113307

**Published:** 2020-06-10

**Authors:** Caroline König, Ahmed Mohamed Helmi

**Affiliations:** 1 Department of Computer Science, Universitat Politècnica de Catalunya, UPC BarcelonaTech, 08034 Barcelona, Spain; 2 ISA DATA S.L, Molins de Rei, 08750 Barcelona, Spain; 3 Department of Computer and Systems Engineering, Faculty of Engineering, Zagazig University, Zagazig 44519, Egypt; amhm162@gmail.com

**Keywords:** condition monitoring, hydraulic systems, sensor signals, convolutional neural networks, classification

## Abstract

Condition monitoring (CM) is a useful application in industry 4.0, where the machine’s health is controlled by computational intelligence methods. Data-driven models, especially from the field of deep learning, are efficient solutions for the analysis of time series sensor data due to their ability to recognize patterns in high dimensional data and to track the temporal evolution of the signal. Despite the excellent performance of deep learning models in many applications, additional requirements regarding the interpretability of machine learning models are getting relevant. In this work, we present a study on the sensitivity of sensors in a deep learning based CM system providing high-level information about the relevance of the sensors. Several convolutional neural networks (CNN) have been constructed from a multisensory dataset for the prediction of different degradation states in a hydraulic system. An attribution analysis of the input features provided insights about the contribution of each sensor in the prediction of the classifier. Relevant sensors were identified, and CNN models built on the selected sensors resulted equal in prediction quality to the original models. The information about the relevance of sensors is useful for the system’s design to decide timely on the required sensors.

## 1. Introduction

Condition-monitoring (CM) is a data driven approach for the supervision of the working conditions of machines and for the detection of failures in an early stage. Based on sensor data, which reflect the physical condition of the machine, the machine’s health state is predicted [1]. Conventional condition monitoring in industrial automation is based on rigid preestablished rules and thresholds, which have a limited capability to recognize failures in complex multisensory systems. Computational intelligence and machine learning (ML) are promising solutions for smart condition monitoring in real-world industrial applications [2], since these methods can learn from historical data and are suitable for processing high dimensional data from multiple sources. Condition monitoring itself is an interesting application in industry 4.0, as it allows to detect automatically incipient machine failures [3]. It is, therefore, an enabling technology for condition-based maintenance (CBM), an industrial predictive maintenance paradigm, which allows to schedule maintenance operations on an on-demand basis. The CBM approach benefits by lower operational costs, reduced machine downtimes and also higher levels of security and safety in the operations of machines since major breakdowns with potential harmful impact on environment and humans are avoided [4].

In recent years, many investigations have reported highly efficient condition monitoring systems based on deep learning models for different industrial applications [5]. Interesting examples are the monitoring of wind turbine gearbox bearing [6] or the supervision of the wear condition in a computer numerical control milling machine cutter [7]. Both authors present a deep learning architecture built on convolutional neural networks (CNN) [8] and long short-term memory networks (LSTM). CNNs implement feature extraction and dimensionality reduction, while LSTMs track the temporal evolution of the signal and predict the observed variable. The success of deep learning models in many fields is related to its ability for automatic representation learning of generic features [9]. This ability is especially valuable in industrial applications as the system should be robust to noise from the environment. Another valuable characteristic of deep learning models is the ability to work directly with raw sensor data avoiding the need for an explicit feature engineering by a human expert from the area. Time series data from sensors are alternatively often studied with feature extraction techniques, such as time-domain (mean, variance, kurtosis or skewness), frequency domain or wavelet domain features [10,11].

In this work, we focus specifically on CNNs, because of their capability to learn feature representations from untransformed high dimensional time series data. The convolutional layers can apply nonlinear transformations and yield abstract patterns in a lower dimension discarding irrelevant or redundant information [12]. In industrial applications, CNN models are used to analyze time series signals of sensors exploiting the network’s ability to learn spatial relationships, which in this case correspond to the temporal evolution of the sensor signal. This type of image analysis on sensor data has been used for fault diagnosis on rolling element bearings [13] or in a gearbox monitoring system with vibration signals [14]. The current work follows a similar approach building CNN models on sensor readings from a multisensorial system of a complex hydraulic installation, where several degrees of degradation of the components are observed. The dataset under study is publicly available from the UC Irvine Machine Learning Repository (published by [15]).

The contribution of the present work is the implementation of ML models for the condition monitoring of the hydraulic, cooler, valve and pump subsystems to predict different levels of degradation of components. The CNN models yield prediction accuracies close to those reported in previous works on the dataset [15,16,17], with the difference that the current approach uses deep learning models and processes the raw sensor data without the need for an explicit feature engineering. Moreover, we carry out a sensitivity analysis of the attributions of the sensor readings for the prediction task of the different degradation states [18]. This high-level information about the relevance of the sensors in the monitoring system is used to study the feasibility to reduce the number of sensors for the detection of failures in certain subsystems. The trend of ML based condition monitoring systems towards the deployment on edge devices [19] imposes additional constraints on the design of ML models, such as the requirement of low energy consumption for example. For this reason, an optimization of the number of sensors through feature selection approaches is opportune as data processing is an energy-consuming task [20] and sensorization by itself additional labor in the engineering of industrial equipment [21,22].

The remaining part of the article describes the analysis with CNN models on the hydraulic system dataset. First, the condition monitoring application of the hydraulic system and the dataset are explained in Section 2. Next, the construction of the ML models is described including the data preprocessing step (Section 3), feature representation learning (Section 4) and the ML model construction (Section 5). In the subsequent sections, the classification performance of the model is discussed (Section 6 and Section 7). Section 8 addresses the attribution analysis of the sensors in the prediction of the degradation states using occlusion maps [23] as well as the optimization of the ML models. The article concludes in Section 9 with a discussion about the results and futures lines of works.

## 2. Hydraulic System and Sensors

Hydraulic systems are widely used in industrial applications and condition monitoring is therefore important as faults can happen at the hydraulic component itself, the entire hydraulic drive, or the hydraulic fluid [24]. Cooling water networks are especially relevant and critical in many industrial applications because of their use for overheat protection. Nevertheless, their study is not trivial as these circulating water systems rely on complex hydraulic and thermodynamic models. Forecasting of water temperature is manifold and has been addressed in different applications. Neural network models were applied by [25] for the monitoring of cooling water networks in a petro–chemical facility. An adaptive neuro–fuzzy inference system [26] was used for the prediction of the temperature in a reversibly cooling tower, part of a heat pump system, to avoid water freezing.

In the present work, we focus on the study of a hydraulic system, which was presented by [15,16] as a test bench in the scope of the iCM Hydraulics project. The authors provide public available data from this hydraulic system for the study of characteristic hydraulic system failures based on sensor readings from a set of sensors installed in the circuit.

**Figure 1:**
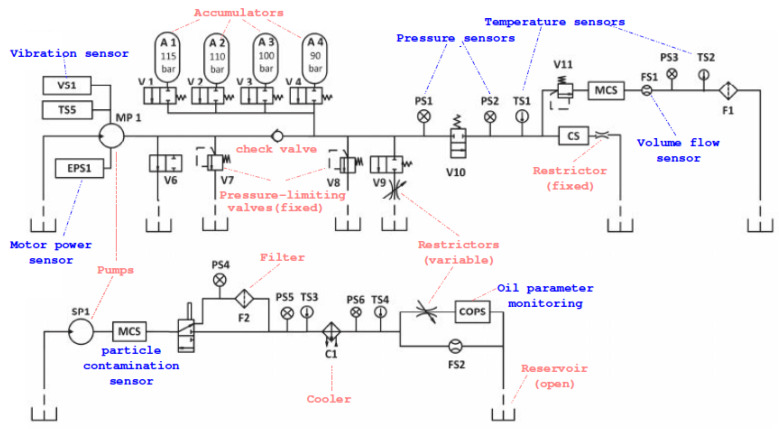
Diagram of the hydraulic test bench (source [15,17]). The upper circuit represents the primary working one which is connected to a lower circuit for cooling–filtration via the oil tank. Sensors are highlighted in blue.

As explained in [27], the hydraulic system (Figure 1) consists of a primary circuit for the load control and two subsystems with the cooling and filtration circuits, which are connected via the oil tank to the primary circuit. Several typical system failures, such as internal pump leakage, pressure leakage in the accumulator, delayed valve switching or reduced cooling efficiency are studied. The authors collect measurements from 17 sensors (Table 1) installed in the test bench and take measurements during work cycles of 1 min. The involved sensors are common industrial process sensors, such as pressure, flow, power, temperature and vibration sensors with a sampling rate ranging between 100 Hz and 1 Hz. Data acquisition is carried out using a PLC (Beckhoff CX5020) device with data transference to a PC via EtherCAT.

The authors provide a detailed description of the components of the test bench and the experiment’s approach to reversibly change the state or condition of the hydraulic system’s components [15]. The approach consists in the simulation of different working conditions during repeated load cycles. A pressure valve (V11) is used to generate variable load levels. In the following the measuring criteria for the simulation of the fault conditions at the different components are described. The main pump (MP) has an electrical motor power of 3.3 kW with a switchable orifice (V9) for simulation of the internal pump leakage. Switching degradation is controlled by the valve’s current (V10) using set-points of its nominal current value according to the intervals of 100%, 90%, 80% and 73%. Gas leakage is monitored via the accumulator (A1–A4) with four precharge pressures of 90, 100, 110 and 115 bar. Cooling power decrease (Cooler C1) is controlled by the fan duty cycle operating in the range of 0.6 to 2.2 kW of power consumption.

Table 2 describes the taxonomy of faults of this experiment with detail. There are four condition monitoring target variables: Cooler (for cooling power decrease), Valve (for switching characteristic degradation), Internal Pump Leakage and Hydraulic Accumulator (for gas leakage). Each target variable has multiple classes, which represent different degradation states of the system.

The dataset comprises sensor readings from multiple sensors. Table 1 shows the details of the measurements of the 17 sensors, which comprise 14 physical sensors—pressure (PS1–PS6), motor power (EPS1), volume flow (FS1–FS2), temperature (TS1–TS4) and vibration (VS1)—and three virtual sensors denoting computed values—efficiency factor (SE), virtual sensors for cooling efficiency (CE) and cooling power (CP). For each sensor, the measurements are recorded during a load cycle of 60 s. As stated in Table 2, the dataset comprises the measurements during 2205 load cycles. In each cycle, the measurements are labeled with a given degradation state for each of the four condition variables. See Table 2 for a summary on the distribution of instances for each condition variable target.

## 3. Sensory Data Preprocessing

**Figure 2:**
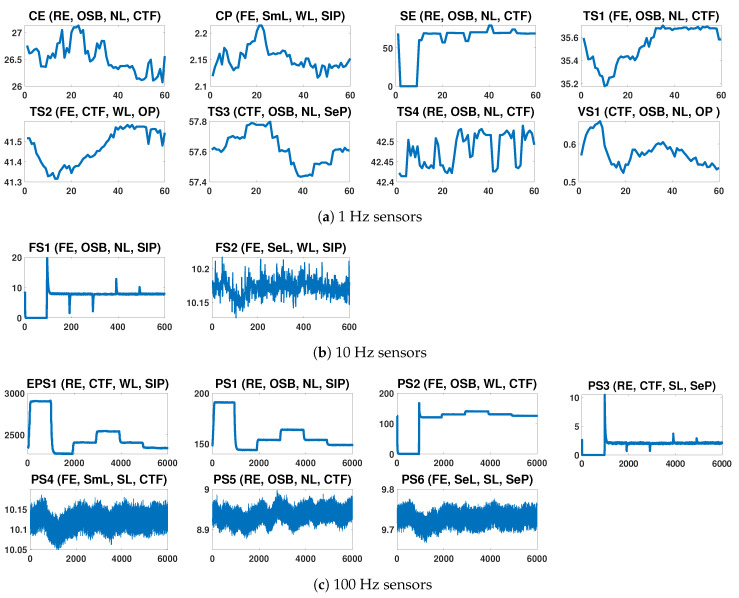
Signals of the 17 sensors of the test bench during selected load cycles. Each subfigure relates to a sensor signal showing the abbreviated sensor name followed by a short description of the degradation state of the four condition variables (cooler, valve, pump, accumulator).

As shown in Table 1, the original dataset contains sensor streaming data from different types of sensors and sampling rates. Figure 2 illustrates the signals of the different sensors of the system during some selected load cycles. For each signal the abbreviated sensor name and a short description of the observed degradation states for each of the four condition variable is shown. For example, the signal Cooler Efficiency (CE) is depicted for a load cycle with Reduced Efficiency in the Cooler, Optimal Switching Behavior in the Valve, No Leakage state in the Pump and a Close to Failure state in the Hydraulic system.

**Figure 3:**
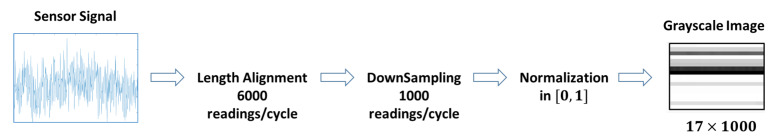
Data preprocessing of sensor signals.

Regarding the sampling rates, pressure and motor power are sensed at 100 Hz, volume flow at 10 Hz, and temperature and vibration at 1 Hz. This mismatch between the sampling frequencies of sensors is a common issue in real-world machine monitoring applications. Preprocessing is needed to obtain complete data readings for all variables at given time intervals. This is important as ML based condition monitoring is synchronous presenting a complete dataset with values for all sensor readings to the ML model periodically. Figure 3 describes the approach of homogenization of the sampling rates for the sensor data.

The first step upsamples sensors readings to the maximum sampling rate of 100 Hz. This implies an increase in the sampling frequency for the volume flow sensors (FS1/2) by 10 and an increase by 100 for the temperature and vibration sensors (TS1–TS4). The result is an aligned dataset with 6000 readings per minute (cycle) for all 17 sensors. As this frequency might be quite high, the dataset is downsampled by taking the average of each six consecutive readings of a sensor yielding a dataset with 1000 values/minute for each sensor. In the following, the data readings of the sensors are normalized in the (0,1) range. The motivation behind downsampling (in fact, only readings of pressure and motor power sensors are affected) is to obtain a more treatable and reasonable image size for the experiments as the training time of a CNN model increases with the image size.

Finally, the sensor readings corresponding to a cycle are transformed into a data matrix of size 17×1000, which is the entry dataset for the deep learning network. The combination of information from different sensors at the data level is referred to as early information fusion of multimodal data [28]. The use of early fusion with several multimodal datasets has been discussed in [29] and followed in [30] in the implementation of a fault detection system in rotatory machines.

## 4. Feature Representation Learning

The contribution of deep learning models in condition monitoring is attributed to its capability to automatically learn representative features from the raw data delegating feature representation learning to the layers of the network [2,5].

In this work, we follow the same strategy using CNNs to learn automatically the temporal relationship of the raw sensor data. CNNs are able to capture spatial relationships in multidimensional data and are high accurate in difficult ML tasks, such as pattern recognition in images and speech analysis [31]. In our work, we focus on leveraging these capabilities of CNNs to recognize automatically temporal patterns in the raw sensor data. For this reason, we apply CNNs to the 2D representations of the raw sensor data. The 17×1000 data matrix is represented as a 2D grayscale image for its evaluation in the CNN model, where each row corresponds to the readings of a sensor during a load cycle. Convolution layers are applied to the data matrix to recognize the spatial relationships and location specific patterns. In this case, 1D relationships are analyzed, as the data are sequential sensor readings.

Alternative deep neural networks for automatic feature representation learning are Autoencoders (AE). AEs are deep learning networks, which use encoding and decoding layers to compress the input features in a lower dimensional latent feature space, so that only the relevant characteristics of the features are kept in the lower dimensional space. AEs are frequently used for the processing of raw sensor signals. Ref. [32] describes AEs for process pattern recognition in industrial processes. Denoising autoencoders (DAEs) are a variant of the AE using random distortion on the input features, so that more robust feature representations are learned by the model, what is especially useful for tackling noise in sensor readings [14].

Finally, a summary of the feature representations used in related work on the dataset is presented in Table 3. Previous works on this dataset relied on explicit feature engineering of statistical features from the time-domain of the signal.

## 5. CNN Model

**Figure 4:**
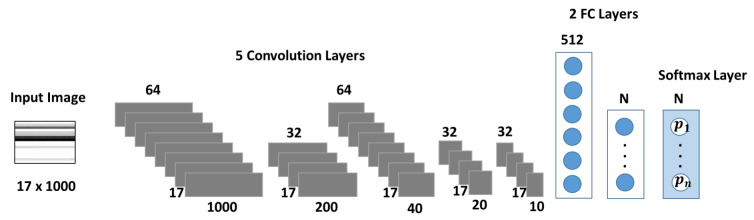
Proposed CNN model. The input image is grayscale with a size of (17,1000) since there are 17 sequences of sensory data. The first and third convolution layer contain 64 filters per layer while the other three layers contain 32 filters per layer. The size of the constructed images by filters in each layer is shown below each layer. The filter size is [1,5] in the first three convolution layers, and [1,2] for the last two layers. Padding is same after first convolution layer and stride is [1,1]. Stride is [1,5] in second and third convolution layers, while it is [1,2] in fourth and last layers. Two fully connected (FC) layers, one with 512 neurons and the second one has a size according to the number of target classes. Finally, there is a softmax layer to predict labels.

The dataset under study contains four independent condition monitoring variables (see Table 2). Figure 4 depicts the CNN architecture used as a multiclass classifier for each condition monitoring target. The network’s design focuses on capturing the temporal correlation of the sensor signals, which are one-dimensional time series. A similar architecture was implemented in [14], where 1D filter banks were used to analyze vibration signals for fault detection in a gearbox. In [34], a medical application for sleep arousal detection, as well CNNs with 1D architectures were used to analyze multi-channel polysomnogram recordings.

The size of the input layer corresponds to the dimension of the sensor data of 17×1000. Each row represents one sequence of sensory data during a load cycle. The network is composed by five primary blocks for feature extraction. Each block is formed by a tuple of {convolution, batch normalization, relu activation} layers. The first and third convolution (convolution) layers have 64 filters, while the other three contain 32 filters in each case. To capture patterns from the temporal evolution of each sensor sequence, convolutions are performed separately on each row of the dataset by using filters (or kernels) with height 1 in all layers, while the lengths varies from 5 in early layers (convolution 1, 2 and 3) to 2 in deeper layers (convolution 4 and 5). In the first convolution layer strides of size [1,1] and a same padding are applied. This combination keeps the constructed filter image after the first convolution layer with the same dimensions as the input image, therefore improving the ability of the second layer to recognize more patterns from the input image. In the subsequent layers, strides with a different horizontal lengths are applied in the filters to downsize the dimensions of the features. For example, the stride of size [1,5] of the second convolution layer downsizes the constructed filter image to a 17×200 format. In consecutive layers, features are further reduced to the dimensions of 17×40, 17×20 and 17×10. Feature reduction with strides is an alternative to pooling layers in CNNs [35,36].

The last three layers of the network implement the prediction stage. The network architecture comprises two fully connected (fc) layers with 512 neurons in the first layer. The dimension of the second fc layer equals to the number of predicted degradation states of the target under study (size 3 for Cooler and Pump, and 4 for Valve and Hydraulic Accumulator). In the final layer, a softmax funcion is used for the prediction of the label (degradation state).

## 6. Classification Results

The dataset of each condition monitoring target has been split randomly into 70% training and 15% validation data for model construction and 15% for the model evaluation on a test dataset. An early stopping criterion is applied during training to prevent overfitting the model. In general, for the considered condition variable targets, the proposed models converge in 30 to 50 epochs. We have tested a range of mini-batch sizes yielding a size of 90 a good compromise between training time and generalization ability. The initial learning rate is 0.001 and a stochastic gradient-descent algorithm with a momentum of 0.9 is used to train the network. The reported classification results for each condition variable target are the average results from repeating 10 times the construction of the CNN model. The experiments have been carried out under Matlab using a CPU i5, 2.6 GHz with 8 GB RAM.

The prediction of each condition variable represents a multiclass classification problem, where degradation states equal to subclasses. In consequence, several classification metrics are used both to asses the quality of the classifier at the subclass classification level and the multiclass classifier level. At the subclass level, Precision (*Prec*), Recall (*Rec*) and Mathews Correlation Coefficient (*MCC*) are used to evaluate the classification performance for each state. *Prec* is a measure of quality as it describes to which extent all predicted positives are true. *Rec* focuses on the completeness of the classifier measuring to which extent all true positives are detected. Precision and recall are both relevant metrics in fault detection as the system should neither give false alarms (assessed by precision) nor dismiss any failure (assessed by recall) [37]. The *MCC* is an overall description of the classifier’s quality taking into account all elements of the confusion matrix and being, therefore, a robust measure for unbalanced datasets [38]. The coefficient takes values from −1 (for complete misclassification) to 1 (for perfect classification) calculating the correlation between the observed and the predicted classification. In condition monitoring, class imbalance is an issue since it is not trivial to obtain representative datasets of potential failure modes or anomalies as equipment manufacturers are not willing to operate machines until the run to failure state [39]. At the condition monitoring test level, the performance of the multiclass classifier is assessed using the classification accuracy (*Accu*), which measures the proportion of correctly classified instances, and the multiclass *MCC* [40].

In the following, we describe the classification results for each condition variable target by reporting the precision, recall and MCC at the subclass level and accuracy and MCC for the multiclass classifier (See Table 4, Table 5, Table 6 and Table 7). The Cooler target (see Table 4) and Valve target (see Table 5) show a high accurate classification performance both at the condition target test level and for the different states achieving nearly perfect precision and recall measures. In fact, authors in [15] have mentioned in the dataset description that Cooler and Value targets are easy classificable problems, while the other two targets, Pump and Hydraulic Accumulator, are challenging.

In our study, the Hydraulic Accumulator condition achieved an accuracy of 0.98 and MCC of 0.95 (see Table 7). Focusing on the subclass MCC we observe differences in the correct recognition of each state. The Close to Failure state is recognized best achieving a precision and recall of approximately 0.98. The other degradation states, such as the Severely Reduced Pressure and Slightly Reduced Pressure are more difficult to recognize. An interpretation of the respective precision and recall values in both cases show that these systems are expected to detect the correct degradation state with a precision of 0.93 and 0.96, while the event of degradation is recognized to an extent of 0.95 and 0.92 in each case. Regarding the recognition of the Optimal Pressure state, which is not a degradation state, but its recognition might be important for the operation of the installation, the system has a precision of 0.96 for the state recognition and 0.90 regarding the completeness of detection.

In the case of the Pump variable, the overall classification performance is still quite good achieving an accuracy of 0.97 and MCC of 0.91 (see Table 6). The state No Leakage is best recognized achieving an MCC of 0.98 while the recognition of the degradation states Weak Leakage and Severe Leakage is approximately 0.90 regarding precision and 0.90–0.92 regarding completeness.

## 7. Misclassification Analysis

Our experiments revealed differences in the accuracies of the ML models for the condition target variables, which might be related to the complexity of the underlying dataset. The authors of [15] reported also lower classification performance for Internal Pump Leakage and Hydraulic Accumulator. In this section, we focus on the misclassification statistics of these two cases. Table 8 and Table 9 show the confusion matrix of the Hydraulic Accumulator and Internal Pump Leakage condition variable. Results are reported with the average misclassification calculated from five CNN models constructed on a separate train and test set (see Section 6).

Interestingly, these misclassification statistics reveal that the degradation states are confused with those semantically close to them. In the case of Hydraulic Accumulator for example, the misclassification towards the Slightly Reduced or Severely Reduced is not systematic, but with the next or preceding state in the degradation scale. So the state Optimal Pressure is most often confused with the state Slightly Reduced, while the intermediate degradation states Severely Reduced and Slightly Reduced are misclassified towards the closest degradation states. The Close Failure is also most often confused with the Severely Reduced state. The described misclassifications may rely on the similarities in the sensor readings between consecutive degradation states. The analysis of the misclassification cases of the Internal Pump Leakage confirms the same casuistry (See Table 9).

## 8. Sensitivity Analysis of Sensors

Deep learning models are often seen as black-box models as the underlying prediction functions are not easily explainable or interpretable for humans [41]. Due to their capability to implement complex and non-linear relationships in deep network architectures, it is challenging to explain the reasoning of the model, since the network’s parameters, such as weights and transformed features are abstract information. Instead, gradient-based attribution methods are often used for the sensitivity analysis of features in neural networks as their representation is understandable for humans [18]. This approach assigns an attribution value to each input feature of the network, which describes its relevance or contribution to the prediction. The contributions of the features are visualized in an attribution heatmap, “where red and blue colors indicate respectively features that contribute positively to the activation of the target output and features having a suppressing effect on it” [18].

In this section, we present the results of an attribution analysis for the problem under study using occlusion sensitivity maps [23]. This technique falls into the category of perturbation-based approaches, where a certain type of perturbations such as removal, masking or alteration of pixels is carried out in order to measure the difference in the prediction of the target. This information is used to analyze the importance of the features in the prediction of the class employing so-called “partial occluders” for regions in the image.

**Figure 5:**
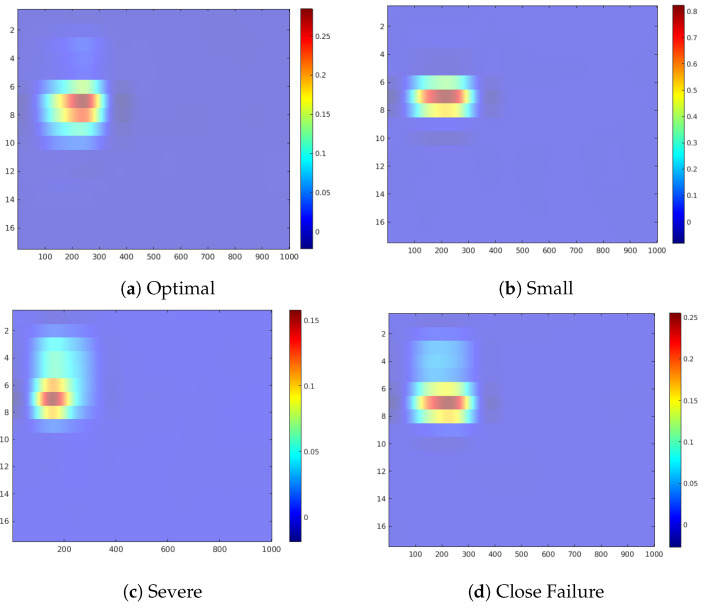
Feature attribution heatmap for the Valve condition.

**Figure 6:**
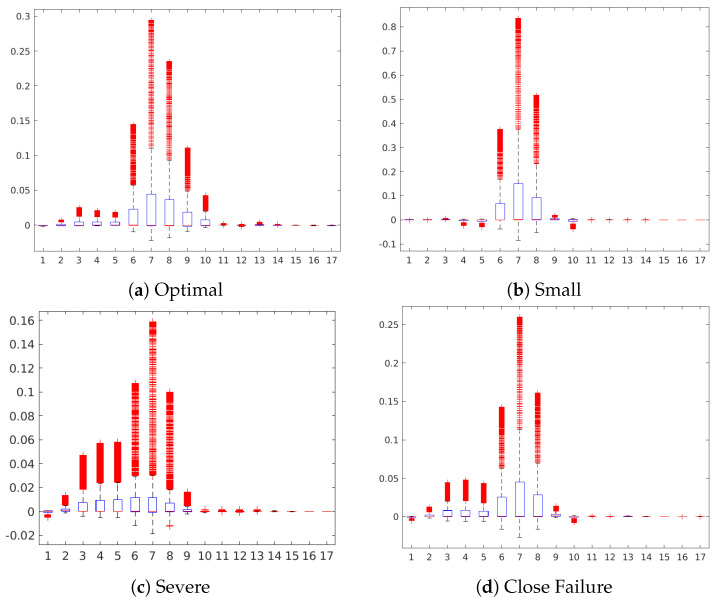
Boxplot of the sensor contributions for the Valve condition.

**Figure 7:**
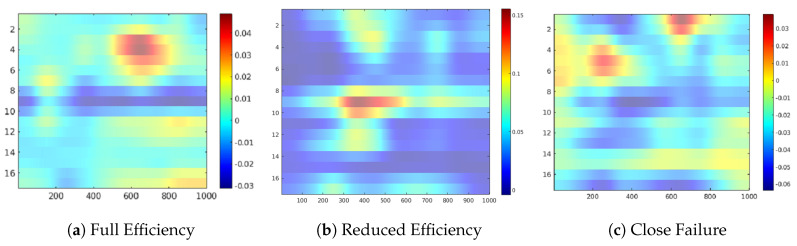
Feature attribution heatmap for the Cooler condition.

**Figure 8:**
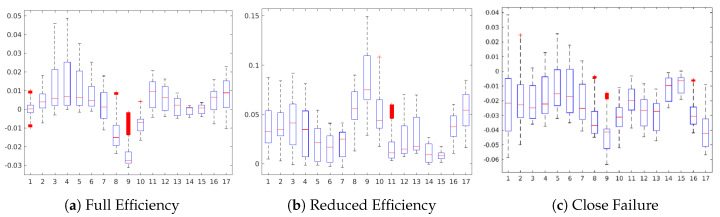
Boxplot of the sensor contributions for the Cooler condition.

**Figure 9:**
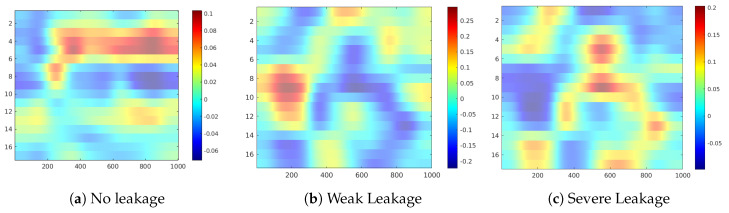
Feature attribution heatmap for the Internal Pump Leakage condition.

**Figure 10:**
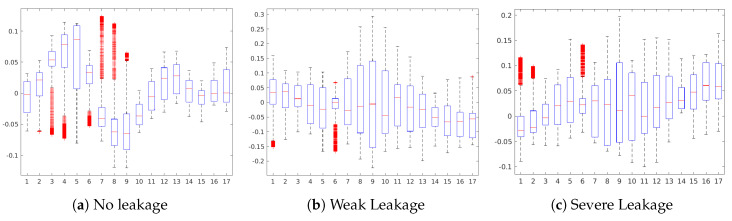
Boxplot of the sensor contributions for the Internal Pump Leakage condition.

**Figure 11:**
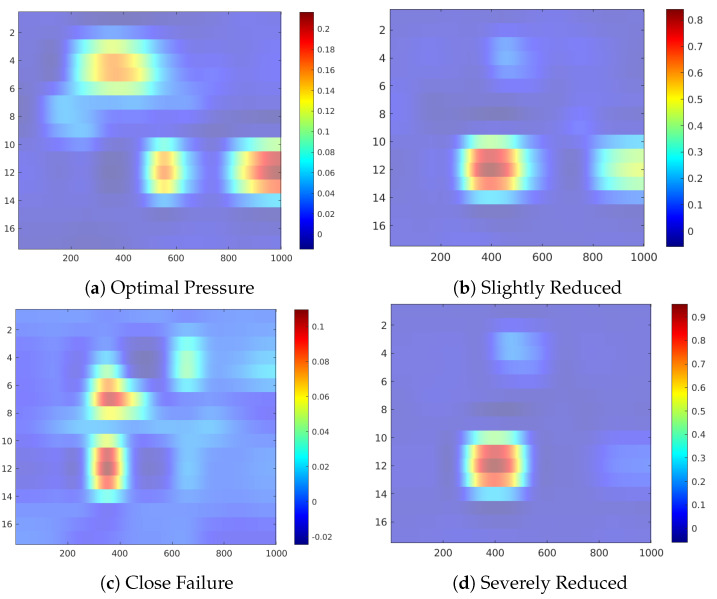
Feature attribution heatmap for the Hydraulic Accumulator condition.

In the following, we discuss the occlusion sensitive maps of the four condition target variables to gain a high-level insight about the contribution of the sensors in the prediction of each state (Figure 5, Figure 6, Figure 7, Figure 8, Figure 9, Figure 10 and Figure 11). The heatmap visualization depicts the absolute value of the contribution to the specified class prediction (red color for positive and blue colors for negative contributions). Such heatmap representations are useful for the visual recognition of the most relevant sensors for the prediction (regions highlighted in red colors) and reveal sensor combinations with common patterns in the contributions. Note that a sensor can have both positive and negative contributions along the sequence of 1000 measurements. Table 10 shows a statistical summary of the maximum attributions in each map by a sensor. The attributions are shown as relative values in a range from 0.0 to 1.0 about the maximum positive or negative contribution of the map in each case. Additionally, we also show a boxplot to give a complete description of the variation in the contributions of a sensor. This statistical analysis aims to show to which extent a sensor is contributing positively/negatively to the prediction of the target.

Figure 5 shows the occlusion sensitivity maps of the Valve target for the different degradation states. The sensor patterns with positive contributions to the prediction of the states remark all similar regions highlighting especially the contribution of sensors #6 to #9 (pressure sensors). The similitude of the maps indicate that similar sensor combinations are relevant for the predictions hinting that the absolute value of the sensor readings may be decisive for the prediction of the state rather than different combinations of sensors. Namely, pressure sensors P1, PS2, and PS3 are the sensors with the highest positive contribution in the prediction of all three states according to the boxplot representation (Figure 6) of the attributions and statistical summary reported in Table 10.

The occlusion sensitivity maps of Figure 7 highlights the relevant sensor readings for the Cooler target. In this case, more complex sensor combinations than those of the Valve target are visible in the map suggesting that a higher number of sensors are relevant for the prediction of the degradation states. Different areas of the image are highlighted for each case. Interestingly, the activation pattern of Reduced Efficiency appear to be complementary to those of Close Failure (See Figure 7b,c). This finding points toward a good separability of the states in the CNN as different combinations of features are involved in the prediction of each state. A more detailed analysis of attributions from the boxplot representation of image of Figure 8 reveals that sensors #8, #9, and #10 (pressure sensors PS3–PS5) are contributing positively in Reduced Efficiency while they have a suppressing effect in Full Efficiency and Close Failure. For the Full Efficiency state many positive contributing sensors are reported, where sensors #3 to #5 (motor power EPS1 and volume flow FS1/2) are important according to the sensitivity map (see Figure 7a). The prediction of the Close Failure states seems to involve a complex combination of sensor readings since the sensitivity map describes mainly negative contributions from almost all sensors (see Figure 7c). Nevertheless, sensors #1 and #2 (cooling efficiency CE and cooling power CP), as well as sensors #5 and #6 (flow sensor FS2 and pressure sensor PS1), reveal some minor positive contributions for this target.

Figure 9 shows the occlusion sensitivity maps of the Internal Pump Leakage target for states No Leakage, Weak Leakage and Severe Leakage. For all three cases, complex combinations of sensor readings are contributing to the prediction of the respective states, where positive and negative contributions are alternated along with the 1000 sensor readings. An analysis of the occlusion map of No Leakage (see Figure 9a) and the corresponding boxplot (see Figure 10a) reveals a positive contribution of the sensors #3 to #6 (motor power EPS1, flow sensors FS1/2, pressure sensor PS1) for the prediction of the No Leakage state. Regarding the occlusion maps of Weak Leakage and Severe Leakage (see Figure 9b,c), the maps show that the activation patterns of these states are complementary, as the positive contributing regions in each case are not contributing in the prediction of the other state. This attrribution hints towards a clear separability between the states Weak Leakage and Severe Leakage in the CNN model.

**Figure 12:**
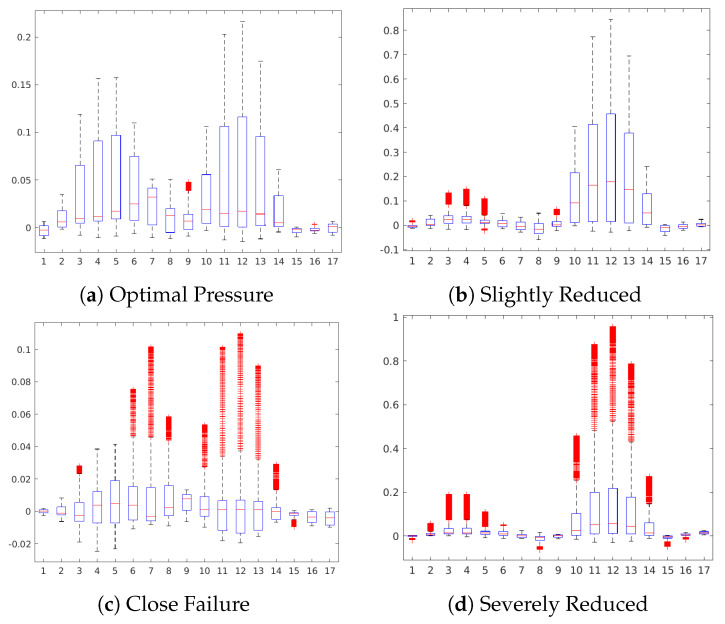
Boxplot of the sensor contributions for the Hydraulic Accumulator condition.

Figure 11 shows the occlusion sensitivity maps for the Hydraulic Accumulator target, while Figure 12 depicts the distribution of the attribution values of each sensor for the prediction of the different degradation states. The sensitivity maps of Optimal Pressure and Close Failure (Figure 11a,c) highlight a group of sensors, therefore revealing more complex sensor patterns involved in these predictions. The readings from sensors #11 to #13 (pressure sensor PS6, efficiency factor SE and temperature sensor TS1) are highlighted in both cases, while for the prediction of Optimal Pressure sensors #4 and #5 (volume flow) are highlighted and for Close Failure sensors #6 and #7 (pressure sensors PS1 and PS2).

According to the results of the attribution analysis, we aim to optimize the ML model by reducing the sensor data to only those sensors marked as highly relevant for the given target. For this purpose, we filter the sensor attributions (see Table 10) by a threshold of θ≥0.5 to select those sensors that are contributing positively at least with 50% in a feature to the prediction of the state. The sensors falling above this filtering rate are highlighted in bold in Table 10. Applying this approach to the four condition variable targets, we can reduce the number of sensors to three for Valve, 12 for Cooler, 16 for Pump and 9 for Accumulator. These findings indicate that solely in the case of Valve and Accumulator a feature reduction is of interest and we decide to build the CNN models for these two targets following the methodology explained in Section 6. For the Valve target we train the CNN model on the sensor readings of the pressure sensors (PS1–PS3). The classification results are the same as those on the complete set of sensors (See Table 5). For the Accumulator target we train the CNN model again with the sensors #3 to #8 (motor power EPS1, volume flow FS1/2, pressure sensors PS1–PS3) and sensors #11 to #13 (pressure sensor PS6, efficiency factor SE and temperature sensor TS1). In this case, the classification results are slightly better than those of the complete set of sensors achieving an MCC of 0.983 and accuracy of 0.993 versus a MCC of 0.982 and accuracy of 0.947 with the entire set of sensors.

## 9. Conclusions

In this study, we have reported the results of a deep learning based condition monitoring application exemplified on a multisensor dataset of a hydraulic installation from the literature. During the development of the solution, we have considered several important aspects of ML based condition monitoring applications, being the prediction quality and interpretability of the model the most relevant aspects.

We have designed a CNN with a 1D architecture to capture the temporal evolution of the sensor signal. The CNN models for each condition variable were close to those reported in previous research on the dataset [17,33]. These previous studies analyzed the performance of several classifiers using time-frequency features and reported results on feature selection and reduction. The main difference of our work regarding those previous studies is the CNN model’s ability to operate directly on multivariate time series data without an explicit feature engineering. Secondly, our work aimed to analyze the classification models at a deeper level. Besides the analysis of classification performance, we also examined the quality of fault detection, detailing the importance of precise and complete detection as condition monitoring systems should neither have false alarms nor dismiss failures. The classifier’s quality was evaluated by the MCC, a robust metric for class imbalance, which is a frequent problem in condition monitoring due to the scarcity of representative datasets of failure modes compared with data from normal operation.

Although the condition monitoring system resulted highly accurate, we have analyzed the misclassifications on the confusion matrix to gain a deeper insight into misclassification patterns. This analysis revealed misclassifications between similar levels of degradation of the condition variable and supported the need to properly characterize faults during the design of a condition monitoring system, as too fine levels of fault detection may difficult the system to operate with high accurate predictions. The proper characterization of faults is a common issue in fault diagnosis. Such a characterization can be done manually on the criteria of an expert of the area or systematically based on the historic failures from a failure database or system log [42] using component breakdown techniques or statistical analysis of failures and downtimes.

In the second part of the work, we focused on the interpretability of the CNN models. The objective was to analyze how each sensor contributes to the prediction of a condition variable in order to optimize the number of sensors. Initially, the CNN models were built on the entire multi-sensory dataset. Attribution analysis through occlusion maps provided useful high-level information about which sensors contribute positively to the prediction of a degradation state. Although for the Cooler and Pump targets, this analysis revealed more complex sensor combinations for the prediction of the condition variable, in the case of the Valve and Hydraulic the identification of relevant sensors was quite straightforward reducing the number of sensors to three and nine respectively. Validation of the sensor selection through the construction of CNN models on the reduced feature set confirmed the adequacy of the feature selection as the classification accuracy remained the same for both models.

The purpose of the here presented study was to showcase how attribution analysis on CNN sensor image data can be used as a feature selection approach for the reduction of sensors during the design of a condition monitoring system. The example of the Valve condition was quite explicative as the attribution analysis highlighted three pressure sensors. In consequence, it would be feasible to center the design of the valve condition monitoring only on a proper sensorization of the pressures of the installation.

As a future line of research, we would like to investigate about the robustness of the CNN model towards missing sensor readings as in certain situations a sensor may not be operative. For example in the case of industrial maintenance, a sensor may need a replacement, which is often not immediately. Meanwhile, the condition monitoring system may operate on incomplete sensor data and should remain operative. This future research would focus on depth completion techniques to learn data representations invariant to missing values either using sparse architectures or special training approaches [43].

## Figures and Tables

**Table 1:** Sensors of the dataset. * CE, CP and SE are virtual sensors.

#	Sensor	Physical Quantity	Unit	Sampling Rate (Hz)	Length Alignment
1	CE	Cooling efficiency *	%	1	×100
2	CP	Cooling power *	W	1	×100
3	EPS1	Motor power	W	100	–
4	FS1	Volume flow	L/min	10	×10
5	FS2	Volume flow	L/min	10	×10
6	PS1	Pressure	bar	100	–
7	PS2	Pressure	bar	100	–
8	PS3	Pressure	bar	100	–
9	PS4	Pressure	bar	100	–
10	PS5	Pressure	bar	100	–
11	PS6	Pressure	bar	100	–
12	SE	Efficiency factor *	%	1	×100
13	TS1	Temperature	${}^{\circ }$C	1	×100
14	TS2	Temperature	${}^{\circ }$C	1	×100
15	TS3	Temperature	${}^{\circ }$C	1	×100
16	TS4	Temperature	${}^{\circ }$C	1	×100
17	VS1	Vibration	mm/s	1	×100

**Table 2:** Summary of the dataset of the hydraulic test bench. A load cycle with a 60 s duration is repeated 2205 times with the distribution of instances as indicated by the examples.

Condition Variable	States	Abbreviation	Examples
Cooler (%)	Close to Total Failure	CTF	732
	Reduced Efficiency	RE	732
	Full Efficiency	FE	741
Valve (%)	Optimal Switching Behavior	OSB	1125
	Small Lag	SmL	360
	Severe Lag	SeL	360
	Close to Total Failure	CTF	360
Pump Leakage	No Leakage	NL	1221
	Weak Leakage	WL	492
	Severe Leakage	SL	492
Hydraulic Accumulator	Optimal Pressure	OP	599
	Slightly Reduced Pressure	SlP	399
	Severely Reduced Pressure	SeP	399
	Close to Total Failure	CTF	808

**Table 3:** Summary of related work. Signal shape features contain slope of linear fit and position of maximum value. Dist. density (1) refers to distribution density characteristics which are median, variance, skewness and kurtosis. In dist. density (2) mean is used instead of median. Classifiers abbreviations are as follows: linear discriminant analysis (LDA), support vector machines (SVM), artificial neural networks (ANN), random forests (RF), naïve Bayes (NB) and linear regression (LR). Interested reader is referred to [17] for more information about PART, JRip, OneR and ZeroR.

Reference	Feature Representation	Features	Classifier
Helwig et. al [15]	Engineered	Signal shape + dist. density (1)	LDA, SVM, ANN
Chawathe [17]	Engineered	Dist. density (2)	RF, J48, PART, JRip, OneR, ZeroR, NB
Quatrini et. al [33]	Engineered	Signal shape + dist. density (1)	ANN, SVM, LR, RF
Proposed approach	Raw sequence data	Encodings of the convolution layers	Fully connected CNN

**Table 4:** Classification results for the Cooler condition at the state and multiclass classifier level.

	Prec	Rec	MCC
Full Efficiency (**FE**)	0.994	0.989	0.987
Reduced Efficiency (**RE**)	1.0	0.998	0.998
Close to Failure (**CF**)	0.989	0.996	0.989
	**Accu**	**MCC**	
**Cooler Condition**	0.996	0.992	

**Table 5:** Classification results for the Valve condition at the state and multiclass classifier level.

	Prec	Rec	MCC
Optimal Switching Behavior (**OSB**)	1.0	1.0	1.0
Small Lag (**SmL**)	1.0	1.0	1.0
Severe Lag (**SeL**)	1.0	1.0	1.0
Close to Total Failure (**CTF**))	1.0	1.0	1.0
	**Accu**	**MCC**	
**Valve Condition**	1.0	1.0	

**Table 6:** Classification results for the Internal Pump Leakage condition at the state and multiclass classifier level.

	Prec	Rec	MCC
No Leakage (**NL**)	0.995	0.990	0.982
Weak Leakage (**WL**)	0.904	0.898	0.871
Severe Leakage (**SL**)	0.904	0.926	0.890
	**Accu**	**MCC**	
**Pump Condition**	0.969	0.914	

**Table 7:** Classification results for the Hydraulic Accumulator condition at the state and multiclass classifier level.

	Prec	Rec	MCC
Optimal Pressure (**OP**))	0.960	0.989	0.965
Slightly Reduced Pressure (**SlP**)	0.958	0.916	0.922
Severely Reduced Pressure (**SeP**)	0.930	0.951	0.927
Close to Total Failure (**CTF**)	0.989	0.981	0.976
	**Accu**	**MCC**	
**Hydraulic Condition**	0.982	0.947	

**Table 8:** Confusion matrix of the Hydraulic Accumulator condition.

	Op	SIP	SeP	CTF
Optimal Pressure (**OP**)	86.6	0.4	0	0
Slightly Reduced Pressure (**SlP**)	3.2	58.2	1.8	0
Severely Reduced Pressure (**SeP**)	0	1.4	54.8	0.8
Close to Total Failure (**CTF**)	0.2	0	1.4	120.2

**Table 9:** Confusion matrix of ther Internal Pump Leakage condition.

		NL	WL	SL
	No Leakage (**NL**)	182.2	0.2	2
	Weak Leakage (**WL**)	0.8	67.2	7.2
	Severe Leakage (**SL**)	0	4.8	66.6

**Table 10:** Maximum attribution by sensor and degradation state.

	Valve				Cooler			Pump			Accumulator			
	OSB	SmL	SeL	CTF	FE	RE	CF	NL	WL	SL	OP	SIP	SeP	CTF
**CE**	−1.00	0.00	0.00	0.00	0.56	0.58	0	0.18	0.54	0.74	0.02	0.00	0.01	0.02
**CP**	0.03	0.00	0.09	0.05	0.48	0.56	−0.19	0.27	0.37	0.63	0.22	0.05	0.04	−0.06
**EPS1**	0.09	0.01	0.29	0.17	0.96	0.61	0.09	0.56	0.14	0.48	0.75	0.16	0.20	−1.00
**FS1**	0.07	0.00	0.36	0.18	0.98	0.54	0.49	0.80	0.21	0.52	0.99	0.18	0.20	0.17
**FS2**	0.06	0.00	0.36	0.17	0.56	0.36	1.00	0.91	0.19	0.63	1.0	0.13	0.12	0.50
**PS1**	0.49	0.45	0.67	0.55	0.56	0.22	0.69	0.55	0.23	0.61	0.70	0.06	0.05	0.81
**PS2**	1.00	1.00	1.00	1.00	1.00	0.19	0.28	1.00	0.59	0.51	0.32	0.04	0.03	1.00
**PS3**	0.80	0.62	0.63	0.62	0.51	0.60	−0.11	0.90	0.87	0.78	0.32	0.04	0.02	0.68
**PS4**	0.38	0.02	0.10	0.05	−1.0	1.00	−0.52	0.53	1.0	1.0	0.30	0.03	0.01	0.24
**PS5**	0.15	0.00	0.01	0.01	0.24	0.73	−0.39	0.04	0.87	0.71	0.30	0.48	0.48	0.07
**PS6**	0.01	0.00	0.01	0.00	0.57	0.40	−0.26	0.00	0.65	0.97	0.55	0.92	0.92	−0.11
**SE**	0.00	0.00	0.01	0.00	0.34	0.47	−0.54	0.23	0.53	0.99	0.61	1.00	1.00	−0.23
**TS1**	0.01	0.00	0.01	0.00	0.13	0.47	−0.70	0.38	0.31	0.75	0.51	0.82	0.82	0.00
**TS2**	0.00	0.00	0.00	0.00	0.09	0.18	−0.33	0.29	0.11	0.40	−0.18	0.29	0.29	−0.91
**TS3**	−0.62	0.00	0.00	0.00	0.14	0.12	−0.20	0.16	0.26	0.77	−0.25	0.00	0.00	−0.25
**TS4**	−0.76	0.00	0.00	0.00	0.36	0.40	−0.80	0.22	0.28	0.71	−1.00	0.02	0.02	0.02
**VS1**	−0.84	0.00	0.00	0.00	0.49	0.57	−1.00	0.43	0.30	0.66	0.01	0.03	0.03	0.04
